# Determinants of mental illness stigma among Tunisian students

**DOI:** 10.1192/j.eurpsy.2023.1845

**Published:** 2023-07-19

**Authors:** M. Ben Amor, Y. Zgueb, A. Aissa, U. Schöberlein Ouali, R. Zaibi Jomli

**Affiliations:** Psychiatry “A”, Razi Hospital, Manouba, Tunisia

## Abstract

**Introduction:**

Mental illnesses affect one in eight people in the world according to the WHO in 2019. They are a leading cause of morbidity and a major public health problem. Stigma harms the quality of life of people with mental illness.

**Objectives:**

Our study aimed to evaluate the association of mental illness stigma with socio-demographic characteristics in Tunisian students.

**Methods:**

This is a cross-sectional study conducted on Tunisian students who anonymously completed a form circulated online through the groups and social network pages related to each academic institution. The form was containing an Arabic validated version of the “Mental Health Knowledge Schedule” (MAKS) and the “Reported and Intended Behaviour Scale” (RIBS) along with a sociodemographic questionnaire.

**Results:**

We have included 2501 Tunisian students with a sex-ratio Male/Female of 0.37. The mean age was 21.57 (±2.55) ranging from 17 to 42 years. Participants’ fields of study were: Science and Technology (58.7%), Literature (17,4%), Economics and management (15.8%), and Arts (4.8%). Among them, 17.1% had a history of family psychiatric disorders and 17.6% had a psychiatric disorder. Besides, 20.9% of the students were using tobacco and 75.6% of them were religious. We also found that 26.7% of participants had previously attended an awareness session. Several determinants had a statistically significant association with the stigma of mental illness in our study population. We noted that females had higher mental health knowledge scores (p=0.001), while males had higher behavior scores (p=0.002). Moreover, students in the scientific and literary fields had higher scores on both MAKS (p<10^-3^) and RIBS (p<10^-3^). In addition, we found greater knowledge of mental illness and less discrimination among participants with a psychiatric history (p=0.013 and p<10^-3^ respectively) and among those who had previously attended a stigma awareness session (p=0.020 and p=0.002 respectively). We also noted higher behaviour scores among people with substance use (p<10^-3^) and lower scores among people with religious beliefs (p=0.009).

**Conclusions:**

Our results show a multiplicity of factors related to mental illness stigma that we can target in anti-stigma strategies. Addressing stigma is a long-term effort; small and large-scale interventions should be considered and evaluated on an ongoing basis to strive for a better future.

**Disclosure of Interest:**

None Declared

